# Supplementary material for: The Effects of Hospitalisation on the Serum Metabolome in COVID-19 Patients
Source: Metabolites. 2023 Aug 16;13(8):951. doi: 10.3390/metabo13080951 (PMC10456321; doi:10.3390/metabo13080951)
Supplement: Supplementary file 1 [file metabolites-13-00951-s001.zip › metabolites-2480546-supplementary figures.pdf]

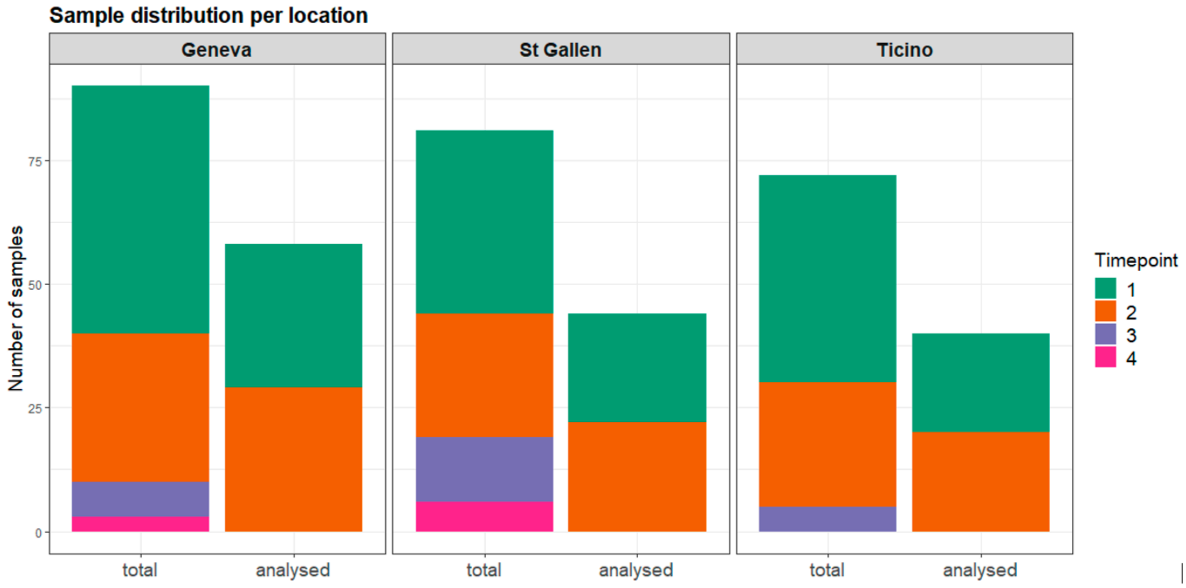

Figure S1: Stacked bar graphs showing the number of patients that had one (green), two (orange), three (purple), or four (pink) samples taken in the raw dataset and in the analysed dataset. Note that the number of analysed samples in timepoint one and two are equal as all patients with a first timepoint but without a second timepoint were removed.

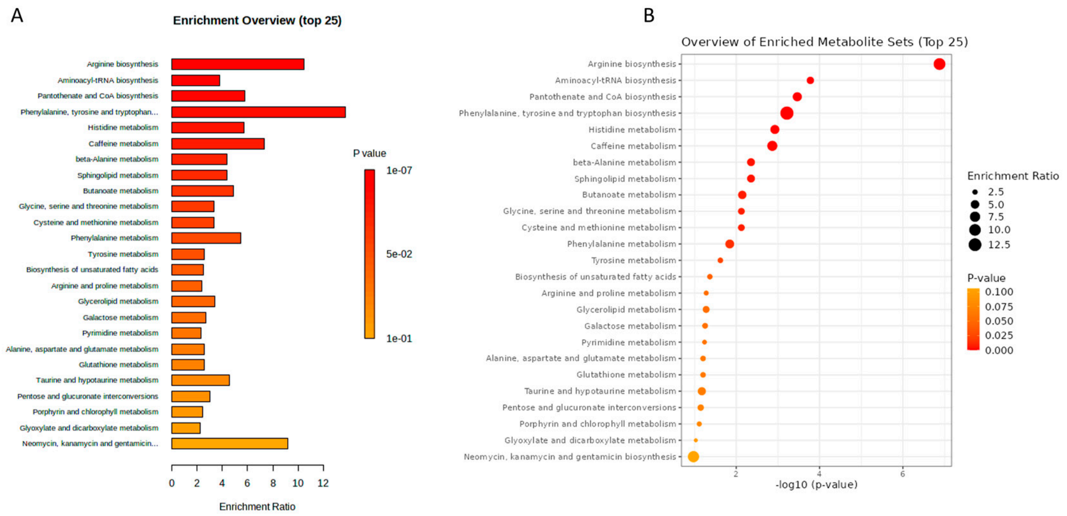

Figure S2: Overview of the top 25 enriched pathways. (A) Bar chart of the enrichment ratios and unadjusted p-values of the 25 most enriched pathways. The enrichment ratio indicates the relative difference between the expected number and the found metabolites in a pathway. (B) Sorted log transformed p-values of the most enriched pathways.

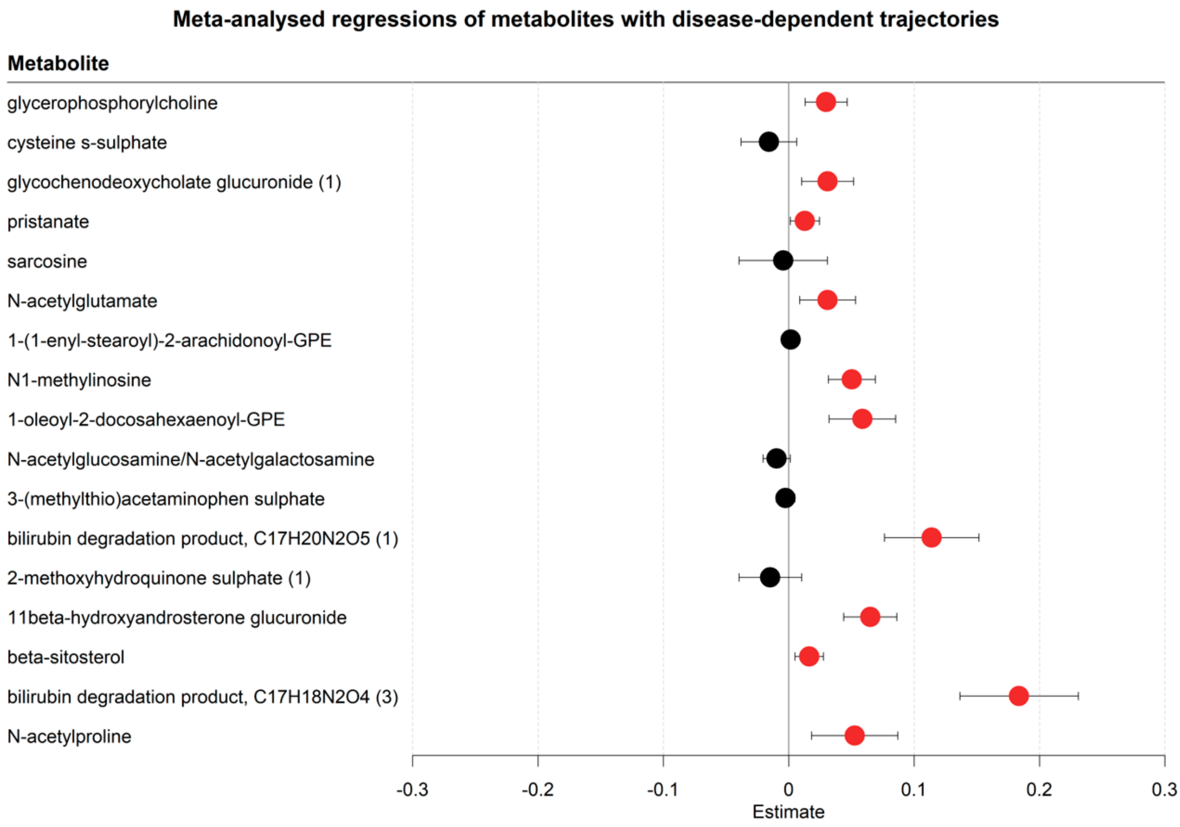

Figure S3: Forest plot on meta-analysed compounds that have differential trajectories between moderate and severe individuals. The estimates, or regression coefficients, represent the pooled change in concentration over time in the three cohorts (see Section 4 for details). Negative estimates indicate decreased serum concentrations, while positive estimates indicate increased serum concentrations during hospitalisation. The displayed metabolites all changed consistently and homogenously between cohorts. Metabolites with red coloured estimates were significantly changed after correction for the false discovery rate. Black coloured estimates indicate no significant change after multiple testing correction. The 95% confidence interval is given by the protruding lines from the metabolite estimate
